# Comparison of Mobile Apps for the Leading Causes of Death Among Different Income Zones: A Review of the Literature and App Stores

**DOI:** 10.2196/mhealth.2779

**Published:** 2014-01-09

**Authors:** Borja Martínez-Pérez, Isabel de la Torre-Díez, Miguel López-Coronado, Beatriz Sainz-De-Abajo

**Affiliations:** ^1^University of ValladolidDepartment of Signal Theory and Communications, and Telematics EngineeringUniversity of ValladolidValladolidSpain

**Keywords:** apps, different income zones, leading causes of death, mobile apps, World Health Organization (WHO)

## Abstract

**Background:**

The advances achieved in technology, medicine, and communications in the past decades have created an excellent scenario for the improvement and expansion of eHeath and mHealth in particular. Mobile phones, smartphones, and tablets are exceptional means for the application of mobile health, especially for those diseases and health conditions that are the deadliest worldwide.

**Objective:**

The main aim of this paper was to compare the amount of research and the number of mobile apps dedicated to the diseases and conditions that are the leading causes of death according to the World Health Organization grouped by different income regions. These diseases and conditions were ischemic heart disease; stroke and other cerebrovascular diseases; lower respiratory infections; chronic obstructive pulmonary disease; diarrheal diseases; HIV/AIDS; trachea, bronchus, and lung cancers; malaria; and Alzheimer disease and other dementias.

**Methods:**

Two reviews were conducted. In the first, the systems IEEE Xplore, Scopus, Web of Knowledge, and PubMed were used to perform a literature review of applications related to the mentioned diseases. The second was developed in the currently most important mobile phone apps stores: Google play, iTunes, BlackBerry World, and Windows Phone Apps+Games.

**Results:**

Search queries up to June 2013 located 371 papers and 557 apps related to the leading causes of death, and the following findings were obtained. Alzheimer disease and other dementias are included in the diseases with more apps, although it is not among the top 10 causes of death worldwide, whereas lower respiratory infections, the third leading cause of death, is one of the less researched and with fewer apps. Two diseases that are the first and second of low-income countries (lower respiratory infections and diarrheal diseases) have very little research and few commercial applications. HIV/AIDS, in the top 6 of low-income and middle-income zones, is one of the diseases with more research and applications, although it is not in the top 10 in high-income countries. Trachea, bronchus, and lung cancers are the third cause of death in high-income countries but are one of the least researched diseases with regard to apps.

**Conclusions:**

Concerning mobile apps, there is more work done in the commercial field than in the research field, although the distribution among the diseases is similar in both fields. In general, apps for common diseases of low- and middle-income countries are not as abundant as those for typical diseases of developed countries. Nevertheless, there are some exceptions such as HIV/AIDS, due to its important social conscience; and trachea, bronchus and lung cancers, which was totally unexpected.

## Introduction

The advances in science and medicine in developed countries have caused an elderly population and long-term survival of individuals who suffer chronic diseases due to modern treatments and cures. This has increased the quality of life expectation of health care consumers [1]. For satisfying this expectation, there have been important improvements in health care delivery supported by the use of the Internet, also known as eHealth, defined by the International Telecommunication Union as the paradigm that encompasses all of the information and communication technologies necessary to make the health system work [2,3]. This paradigm has evolved significantly to the point of creating mobile health (mHealth) as a branch of eHealth.

There are many definitions of mHealth. For some authors it is an area of eHealth that provides health services and information via mobile technologies such as mobile phones and PDAs [4,5]; for others, the term is defined as “emerging mobile communications and network technologies for healthcare systems” [6]. What is clear is that mobile devices are used for providing health care. More important than the definitions are the impressive development and dissemination this field has been achieving, to the point that Atienza et al (2011) have suggested that mobile health may be the “killer app” for cyberinfraestructure for health in the current century [7]. The reality is that important advances in technology and communications have been achieved in the past few years and mHealth has taken advantage of them [8-12]. mHealth is supported by many mobile telecommunications technologies, such as 3G (third generation) or 4G (fourth generation) technologies, for example [13-18].

The potential for mHealth applications is rather well-documented [19-21]; for example, move away from face-to-face visits at the doctor’s office, access to a wide array of educational resources including information on disease-specific topics and general self-management tools, view your own electronic medical record, access information relative to medications, and continuous surveillance of vital or physiological signs.

In addition to this, advances in technology for smartphones and tablets have caused their incredible growth, especially in high-income countries. There were 6 billion mobile subscriptions in 2011 and more than 1.7 billion mobile phones sold in 2012, 712.6 million of which were smartphones. With these numbers, it is obvious that these devices must be used in the field of mHealth to assist every person with one of these gadgets. Indeed, mHealth is already using them as shown by the great number of health applications currently available [22-25].

**Figure 1:**
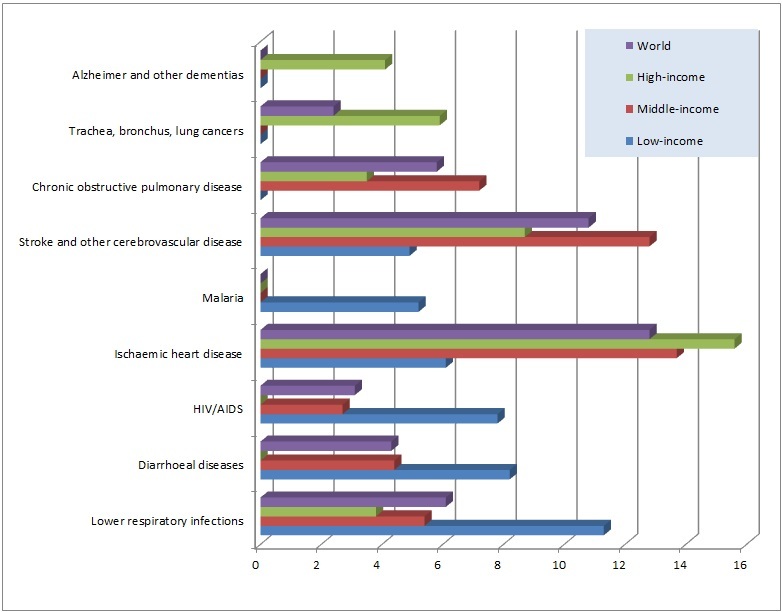
Percentage of deaths caused by the leading causes of death grouped by zones.

These devices can be especially useful for the prevention and management of those diseases that cause high rates of mortality. The World Health Organization (WHO) estimated a total of 56.8 million deaths in 2008 and, excluding 5.1 million that were caused by injuries, the remainder were produced by diseases and health conditions [26]. Some of the leading causes of death are presented in Figure 1, which shows the percentage of deaths caused by these diseases and distributed according to different income zones [27].

The 6 leading causes of death for each zone and worldwide in 2008 are shown. When these diseases have 0% deaths represented in determined zones of Figure 1, it means that the diseases are not among the top 10 in this zone, but not that there are not deaths caused by those illnesses. Some data are presented below.

Considering randomly 1000 individuals dead in 2008, statistically 159 would have come from high-income countries, 677 from middle-income countries, and 163 from low-income countries [28]. Cardiovascular diseases (CVDs) are the deadliest diseases—17.3 million people died from CVDs in 2008, representing 30% of all global deaths [29]. Among these diseases, ischemic heart disease (IHD) is the leading cause of death globally with an estimated 7.3 million deaths [30-34]. Stroke produces not only death, but also disabilities and high probabilities of death in the future. Its burden is projected to rise from approximately 38 million DALYs (disability-adjusted life years) worldwide in 1990 to 61 million DALYs in 2020 [35-38].

Lower respiratory infections (LRI) are the leading causes of child mortality in the world, producing 1/5 of mortality in children under 5 years. The respiratory syncytial virus (RSV) is the single most important cause of severe respiratory illnesses in children and can provoke pneumonia, which causes 90% of these deaths due to the virus [39-43]. Sixty-five million people had chronic obstructive pulmonary disease (COPD) and more than 3 million died in 2005 [44-48]. Diarrheal disease is a major problem in developing countries and the second leading cause of mortality in children under 5 years of age, killing 1.5 million children every year [49-52].

Human immunodeficiency virus/acquired immunodeficiency syndrome (HIV/AIDS) is a major global public health issue. In 2011, there were 34 million people living with HIV and 1.7 million of whom died because of it. The political commitment, social mobilization, and HIV/AIDS funding done by almost every country in the past years have contributed to the total of 95 million people tested in 2010 and more than 8 million receiving antiretroviral therapy [53-57]. According to the WHO, there were 219 million cases of malaria and 660,000 deaths in 2010 [58-60]. However, other studies have worse numbers: Murray et al (2012) estimated 1.24 million deaths globally in 2010 [61].

Cancer caused 7.6 million deaths worldwide in 2008, with the majority caused by lung cancer (1.37 million deaths). Tobacco is the most important risk factor for developing cancer (not only lung cancer), causing 22% of cancer deaths and 71% of lung cancer deaths [62-65]. There are 35.6 million individuals suffering from dementia and 477,000 annual deaths worldwide. Among the different dementias, Alzheimer disease is the most common with a contribution of 60%-70% of the cases and a median life expectancy of 7.1 years [66-70].

The main aim of this paper is to continue the research begun by the authors about mobile apps for the most prevalent health conditions [71], focusing on the diseases and conditions that are leading causes of death by the WHO grouped according to different income regions [27]. For this purpose, two reviews have been done. The first was a literature review carried out by searching published articles in several systems, and the second was a review of commercial apps done in the most important mobile phone apps stores considering the market share of the operative systems used for smartphones. The main objective is to find out which diseases are more researched and which have more apps, comparing these findings with their weight in mortality, not only globally but also distributed according to different income regions. This study is only limited to a general search of applications, without studying or analyzing them due to the significant extension of that work, which is enough for additional research.

## Methods

### Overview

In April 2013, two reviews were developed: a literature review and research in commercial applications stores. The procedures used for each review are explained below.

### Literature Review

For the literature review, a search of published papers was developed in the following databases and systems: IEEE Xplore, Scopus, Web of Knowledge, and PubMed. When searching for a specific disease, a combination of search words was used. If the number of results obtained was too low, another combination of words was used until a more significant number of results was obtained. These terms were used on all the systems mentioned. The process was repeated with each disease studied. The search strings were used only for metadata and the article search was limited to the past 10 years, from 2003.

**Table 1:** Terms used in the literature search strings of each disease.

Disease	Term1	Term2
LRI	lower respiratory infections	application
	respiratory infections	application
	respiratory diseases	application/app
Diarrheal diseases	diarrhoeal diseases	application
	diarrheal diseases	application
	diarrhea	application/app
	diarrhoea	application/app
HIV/AIDS	HIV/AIDS	application/app
	HIV	
IHD	ischaemic heart disease	application/app
	ischemic heart disease	application
	heart disease	application/app
Malaria	malaria	application/app
Stroke and other cerebrovascular diseases	stroke	application/app
	cerebrovascular disease	
COPD	chronic obstructive pulmonary disease	application/app
	copd	
Trachea, bronchus, lung cancers	trachea cancer	application/app
	bronchus cancer	
	lung cancer	
	respiratory system cancer	
Alzheimer and other dementias	Alzheimer	application/app
	dementia	

**Figure 2:**
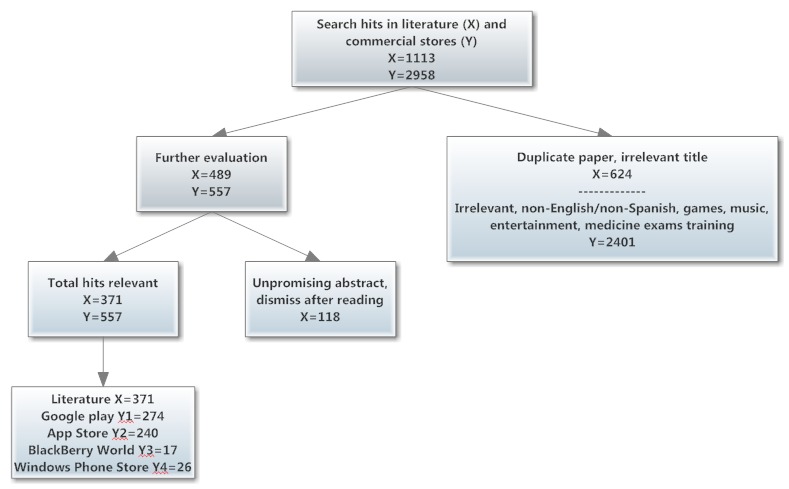
Flow chart of the steps followed in the reviews.

Figure 2 shows a flowchart with the steps followed in both literature and commercial reviews. All the systems returned 1113 results, with 624 repeated or with an irrelevant title for this study. Of the remaining 489 papers, 118 were dismissed after reading their abstract or the whole paper when necessary. Finally, a total of 371 papers (33.3%) were selected as relevant. For considering a paper relevant, it had to fulfil some criteria: it must be focused on applications using mobile phones or devices, it must be written in English, and it has to be about a mobile app or apps designed for the sought condition. This means that papers centered on applications for several and different diseases were dismissed even if one of the illnesses treated was the one sought.

For the search strings, in some cases Britain and US terms for the same word were used to ensure that every relevant document was revealed. The combinations of words used were the following: Term1 AND mobile AND Term2, Term1 AND m-health; Term1 AND “mobile phone”; Term1 AND smartphone; where Term2 was app or/and application and for Term1 it was used the terms used for each disease that is shown in Table 1.

### Commercial Apps Review

The second review was carried out in the most important applications stores of the smartphones industry considering the market share of the operative systems for smartphones [23-25,72]. In descending order of this market share, the stores are Google play of Google Android [73], iTunes of Apple [74], BlackBerry World of BlackBerry [75], and Windows Phone Apps+Games Store of Microsoft [76].

The process is similar to the one followed in the literature review. Table 2 shows the different terms used in the search of the applications related to each disease and the flowchart of Figure 2 shows the steps followed in the commercial review. A total of 2958 apps were initially found although, after checking whether the apps were relevant to the study and whether some conditions were fulfilled, only 557 (18.8%) met these requirements. The requisites to include an app in the study were applications not in English or with the description in a different language from English or the one of the country where the search was done (Spanish) were dismissed, the same as those included in the categories of games, music, or entertainment. Applications that use flashcards for helping medical students in their exams and applications for conferences were also dismissed.

**Table 2:** Strings used in the search of commercial apps for each disease.

Disease	Search String
LRI	respiratory infections
Diarrheal diseases	diarrheal disease
HIV/AIDS	HIV
IHD	“heart disease”
Malaria	malaria
Stroke and other cerebrovascular diseases	stroke; “cerebrovascular disease”
COPD	”chronic obstructive pulmonary disease”; copd
Trachea, bronchus, lung cancer	“trachea cancer”; “bronchus cancer”; “lung cancer”
Alzheimer and other dementias	alzheimer; dementia

## Results

### Mobile Apps in Literature

The results of relevant papers for each condition and each system are presented in Table 3. The last column shows the total number of different papers found on all the systems. The results of the search of the respiratory system cancers can be broken down into nine results for lung cancer and no results for the rest of the search strings (trachea cancer, bronchus cancer, and respiratory system cancer).

In light of the results, heart diseases are the most researched ones. It is followed by HIV/AIDS, Alzheimer and other dementias, and stroke and other cerebrovascular diseases. LRI and COPD hold the fifth and sixth places, respectively, in descending order of research done and the last places are for malaria, trachea, bronchus, lung cancers, and diarrheal diseases with very little investigation; only 28 articles among them.

The majority of papers found were relative to the design, development, or implementation of mobile systems, whole systems [77], complement systems to a mobile phone [78], or part of the system [79]. There are also evaluations and validations of these mobile systems [80]. Other types with a great number of papers found are interventions using mobile systems [81], apps [82], or mobile phones [83] and the studies and evaluations of these interventions [84]. Another type of paper found, but less frequent, are those dedicated to applications for smartphones, with add-on complements [85] or without them [86] and reviews of the existing apps for a specific objective [87].

**Table 3:** Results of the literature review.

Disease	IEEE	Scopus	Wok	PubMed	Total
LRI	9	21	9	3	24
Diarrhoeal diseases	0	3	1	0	3
HIV/AIDS	4	77	38	42	86
Heart diseases (IHD)	41	81	47	32	121
Malaria	1	15	9	8	16
Stroke and other cerebrovascular diseases	7	30	13	9	36
COPD	5	20	10	7	23
Trachea, bronchus, lung cancers	2	7	4	3	9
Alzheimer and other dementias	16	49	20	7	53

### Mobile Apps in Stores

The findings of the commercial apps review are revealed in Table 4. Each cell shows the number of relevant apps out of the total number of results found in each commercial store. The last row contains the addition of the applications found for all the diseases at each store and the last column presents the addition of the applications found at all the stores for each sought disease. Nevertheless, this number does not represent the total number of different apps in all the stores, because there are apps developed by the same creator for different operative systems, being the same (or similar) app for different smartphones software. For example, the application AIDSinfo HIV/AIDS Glossary, created by the National Library of Medicine at National Institutes of Health (NIH) [88], is available on iTunes [89] and on Google play [90].

There are some diseases issued by the WHO that are actually a group, so in Table 4 these groups are divided into its illnesses. This way, the groups stroke and other cerebrovascular diseases and Alzheimer and other dementias are divided each one into two rows, one for stroke and cerebrovascular diseases and another for Alzheimer and dementia, respectively. The same occurs with the group trachea, bronchus, and lung cancers, split into three rows corresponding to the three types of cancer.

Focusing on the number of applications for each disease, the ones with more apps are Alzheimer and other dementias with 128, followed closely by HIV/AIDS. The third position is for heart diseases with 111 applications and the fourth is for stroke and other cerebrovascular diseases despite the fact that there are no results for cerebrovascular diseases. After a gap of more than 40 apps, COPD holds the fifth position and trachea, bronchus, and lung cancers the sixth, although there are no apps for the two first mentioned cancers, only for lung cancer. The seventh and eighth positions are malaria and diarrheal diseases, and the last is LRI with only 6 apps.

**Table 4:** Results of the commercial apps review.

Disease	Google play	iTunes	BlackBerry World	Windows Phone Store	Total
LRI	5/57	1/17	0/0	0/0	6
Diarrheal diseases	12/88	8/12	0/0	0/5	20
HIV/AIDS	54/238	56/121	7/8	7/19	124
Heart diseases (IHD)	60/249	44/79	3/4	4/21	111
Malaria	12/51	7/25	1/2	2/8	22
Stroke	27/480	45/530	4/46	3/105	79
Cerebrovascular diseases	0/0	0/2	0/0	0/0	0
COPD	20/50	17/37	0/0	1/3	38
Trachea cancer	0/0	0/1	0/0	0/0	0
Bronchus cancer	0/1	0/0	0/0	0/0	0
Lung cancer	14/88	14/54	1/3	0/2	29
Alzheimer	38/175	25/96	1/2	6/14	70
Dementia	32/185	23/70	0/0	3/10	58
Total apps per store	274	240	17	26	

The main types of apps for LRI are guides and calculators for health care professionals, although there are also some informative apps for patients. In the case of diarrheal diseases, the most common apps are natural and personal remedies as well as some guides. Referring to HIV/AIDS, there are many guides for health care professionals, patients and public in general, centering, in this case, on educational aspects. There are also a significant number of apps with news regarding HIV/AIDS. The great majority of apps for heart disease are heart rate monitors for patients and algorithms and calculators for specialists [91]. The principal focus of malaria apps is to use the smartphone as a mosquito repellant, followed by informative apps about it. The most usual apps for stroke and other cerebrovascular diseases are stroke detectors and stroke scale calculators. There are also some informative apps.

Referring to COPD, the majority of apps are informative and guides for health care professionals, followed by some COPD trackers and apps for learning to use inhalers, both destined for patients. Focusing on lung cancer (there are no specific apps for the rest of cancers studied), most apps are destined for cancer stage determination and help in its diagnosis for professionals. For patients, informative apps are common. Finally, typical apps for Alzheimer and other dementias are games useful for their prevention, supportive apps for patients and relatives, as well as trackers and apps for auto-checking the status of a dementia.

**Figure 3:**
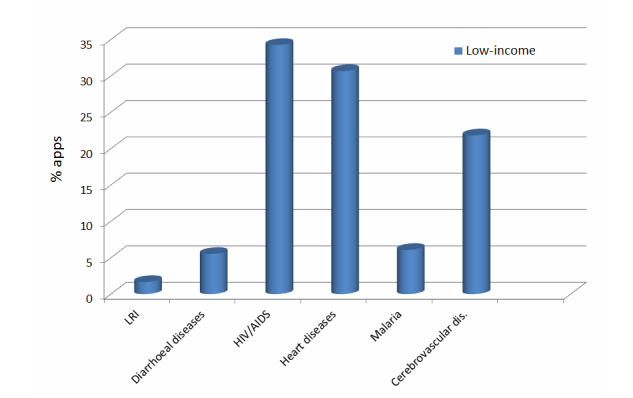
Percentage of apps (%) related to the top 6 causes of death of the low-income zone.

**Figure 4:**
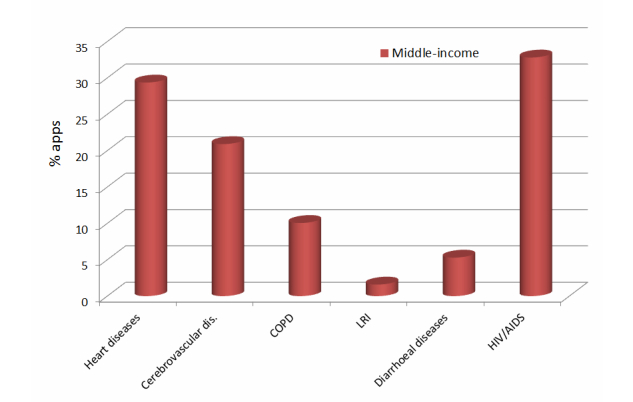
Percentage of apps (%) related to the top 6 causes of death of the middle-income zone.

**Figure 5:**
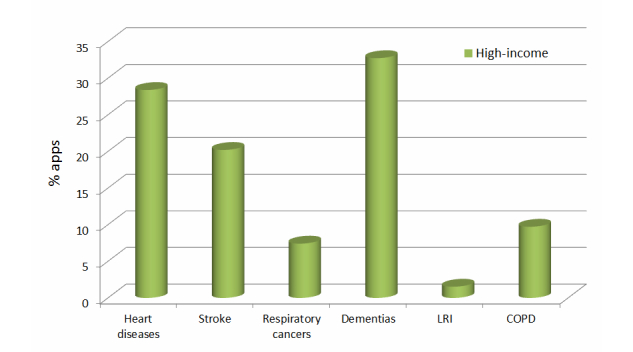
Percentage of apps (%) related to the top 6 causes of death of the high-income zone.

Figure 3-5 show three graphs, which present the percentage of the number of apps found in the applications stores for each of the top 6 causes of death for each region, excluding the rest of the total (100%).

The diseases with a higher percentage of apps in low-income and middle-income countries are heart diseases (IHD) and HIV/AIDS, whereas in high-income countries these diseases are Alzheimer and other dementias, and heart diseases (IHD). LRI is the disease with the least percentage of apps in all the zones, followed by diarrheal diseases in low- and middle-income countries and trachea, bronchus, and lung cancers in high-income countries.

## Discussion

### Principal Findings

Some important conclusions can be obtained from the analysis of the results. Comparing the numbers of the literature review with the numbers of the commercial apps review, it is clear that there is more work done in the commercial field than in the research field. This is quite logical because the main objective of developers is earning money with their apps and, therefore, they focus on commerce. However, to create a good application, it is necessary to do some research [92], and this research can be a market study and/or an investigation about the application itself, including aspects such as the type of application, the necessity to cover, or the target public, among others. Unfortunately, what can be extracted from the results of this study is that most developers only do the market investigation or they do not publish the results of their studies while developing their apps. Contrasting the literature review with the commercial review, there are two differentiated groups. On one hand, there are four diseases with the highest percentage of work done in developing research and applications. These diseases are Alzheimer and other dementias, heart diseases, HIV/AIDS, and stroke and other cerebrovascular diseases. The numbers of the commercial apps review for the first three diseases mentioned before are similar and over 100 apps while for stroke and other cerebrovascular diseases the number of apps is much higher than the rest, which enforces the idea of two groups. On the other hand, the second group is formed by the rest of diseases, having much less work done in both research and commercial fields.

Another piece of information extracted from this comparison is that the position held by a disease in number of apps or papers found is similar in both reviews, with a maximum difference of two positions except for LRI with the fifth and ninth positions in the literature and commercial reviews, respectively. Hence, for the majority of the studied diseases, there is a concordance in the proportion of work done for each disease in both fields, which means that both researchers and developers generally agree on the importance given to each disease.

Contrasting the order of diseases according to the number of apps and papers and by mortality worldwide, there are some interesting issues. The most striking is that the diseases with more commercial apps, Alzheimer and other dementias, are not included in the top 10 causes of death worldwide. Therefore, why is there so much effort and work done in these illnesses? As exposed below, the answer is easy: Alzheimer and dementias are typical of high-income countries [27] where there is a social conscience of these illnesses and the population is very much aware of the consequences and dysfunctions that they cause, as shown by the great number of associations worldwide [93,94]. This reason, added to the facts that the majority of developers and developers groups are located in these countries [95-97] and designing apps for richer zones is more profitable, explains this issue.

The contrary occurs with LRI. Worldwide, this disease is the third leading cause of death, but the reviews carried out in this study show that its numbers do not correspond with this position (it is the fifth disease more researched and the last in commercial stores). This can be explained by several reasons: the first is that it is the first cause of death in low-income countries but it is not among the top 3 in middle- and high-income countries and, because developers are generally aware of these countries [95-97], this disease is not a priority for them. However, the previous reason alone is not enough to explain this case because LRI is the fourth and the fifth cause of death in middle- and high-income countries, respectively, above others such as HIV/AIDS, which has more apps and research.

Another important reason is that this disease is typical of children younger than 5 years old [40] and, as a result, developers have no reasons to create apps for LRI patients. One option could be the design of apps for parents, although people in developed zones are not very aware of this disease as they are with other illnesses such as HIV/AIDS [54,98] or diabetes [99], not giving it the importance it really deserves so it is underinvestigated. In addition, in developed zones, children are vaccinated against RSV [42], the most important cause of LRI. Thus, the combination of the previous reasons explains this situation.

There are other similar cases to the previous two, but not so extreme. Hence, as happens with Alzheimer, HIV/AIDS holds the second position in both reviews but it is only the sixth cause of death [54,98]. On the other hand, diarrheal diseases hold the fifth position in mortality but only the eighth and the last position in the commercial and literature review, respectively, quite similar to the case of LRI but more obvious, due to the fact that it is the second leading cause of death in children younger than 5 years old [49] and, in this case, it is not among the deadliest diseases in high-income countries [26,27].

### Limitations

This study presents some limitations in the methodology followed for each review, typical for this type of revision [100]. The process of extracting the data presented a significant risk of uncertainty. Occasionally, the inclusion of a paper or an app in the study is not easy because the text is not clear and it can be misunderstood. To avoid this possible error, we enhanced the assessment process with independent verification. Thus, one author developed the search of literature papers, a second author developed the commercial review, and a third author inspected the results to check for possible errors.

The search results of the literature review were restricted to the past 10 years, from 2003 to the present day. This restriction did not affect the study because before 2003 there was a rather low number of smartphones and mobile devices, only dedicated to business, without commercial stores and health care applications as we know them today [101,102]. In addition, only papers dedicated exclusively to the disease searched were included in the study. Hence, there are papers dedicated to several diseases that can contain interesting information regarding the diseases studied dismissed.

Some limitations were found and addressed (if possible) in the commercial review. The results of the search of respiratory infections included some infections not specific to the lower respiratory system; hence, a discrimination of these results was necessary to select apps specific for lower respiratory infections.

Searching on iTunes, diarrheal disease provides no results, so in this case diarrhoea and diarrhea were used as search strings. The same was tried in the search of this disease on BlackBerry World but it returned no results. Because the disease name is so specific, no other terms could be used to obtain more results relative to it.

In the search of IHD, “ischemic heart disease” returned only 48 apps, very few for the most important cause of mortality. Hence, it was resolute to search only “heart disease” even though IHD is a specific type of heart disease. With this combination of words we found 353 apps, a much more significant number of apps. In addition to this, apps of diets or recipes were not included in the study, but those dedicated exclusively for cholesterol management were included because high cholesterol is a cause of heart malfunctions.

When inspecting the stores about trachea, bronchus, and lung cancers, apps for treatment or assistance in general cancer management that includes the searched cancer were taken into account. However, apps for quitting smoking were not studied despite the fact that smoking is the most important cause of lung cancer. We made this decision because there are other motivations to quit smoking, not only lung cancer, and those apps are not centered on the cancer itself.

In the case of Alzheimer and other dementias, there were a number of games designed for their prevention, although, as mentioned before, only those not included in the category of games were valid for the study (medical, health category). We did this because the majority of the found games included in the category games were not specifically designed for Alzheimer prevention and only took the commercial advantage of saying that they were useful for this purpose.

There were two issues with Google play. The first was that, in some searches, the store indicated that a certain number of results have been found but, while exploring the results pages, the last were blank. Because the number returned by the store and the real number of apps were different, we decided to use the last one, the number of applications shown. The other issue was that, when searching for stroke, the store returned more than 1000 results but only 480 apps were shown. In this case, the number used was the second one, 480. This last issue could affect our study because there are probably more apps apart from the 480 shown by Google. The current version of the store does not present these problems.

Another important limitation in the commercial review was the language used for the search strings. One of the objectives of this paper is to find and compare the existing apps for expressly mortal diseases in different income regions. Ideally, at least the most important low- and middle-income countries languages should have been used to obtain apps only developed in these languages. However, we only focused on the use of English, obtaining all the apps developed for this particular language, because it is the most extended and used worldwide. This barely affected the study of diseases typical of high-income countries because English is commonly used and extended there. Nevertheless, many results were shown in Spanish, because the stores detect the place where the search has been done, showing the apps and their summaries in the language of that place, if possible. In these cases, the apps were also included in the study, if relevant.

It is important to indicate that the search of IHD has actually become a search of “heart disease” in general, due to the lack of results with IHD alone in both reviews. Hence, we will talk about heart diseases instead of IHD. Nevertheless, it does not affect the study, because the applications found are for general heart diseases, including the one searched.

### Conclusions

Comparing the number of apps and research done for disease with the position in cause of death for the three different zones, some interesting conclusions can be extracted. The leading cause worldwide and in middle- and high-income countries, heart diseases (IHD), is one of the most researched and with more apps, a logical fact because developers focus on typical diseases in high-income countries [98-100]. However, there are two diseases, the first and second of low-income countries, which have very little research and fewer commercial applications. These diseases are LRI and diarrheal diseases. The reason is the one mentioned previously: these illnesses are not common (diarrheal diseases) or there is no use of an app for the diseases (LRI), so that their research is not worthwhile, not to mention the fact that they are common in low-income zones where the technology for smartphones and tablets is not widespread [103,104] and, therefore, there are no market opportunities.

Another interesting case is what happens with HIV/AIDS, which is in the top 6 of low- and middle-income zones but even not being among the first 10 causes of death in high-income countries, it is the second disease with more research and applications. In this case, the illness is very popular not only in poor and developing zones but also in richer countries [101] in a way that, despite the fact that it is not one of the worst diseases in mortality in these rich countries, it has the attention of developers (as the numbers of this study show) and the population as a whole. At least, in this case all the countries can feature these apps for HIV/AIDS if they have the means needed.

The most curious case is the one with trachea, bronchus, and lung cancers. They are the third cause of death in high-income countries, but are included in the least researched diseases with regard to apps. The reason is not clear, because cancer in general is a common matter and there is a huge amount of information and applications about it. In fact, the search for “cancer” on the App Store returns 896 apps and 500 on Google play. Moreover, specifically these types have also an important social conscience principally for being consequences of smoking [63,64]. Therefore, it can be considered a good opportunity for developers to fill this empty space in mobile apps for health care.

Focusing on the types of the predominant apps for each disease, there is a common one for all of them: the informative apps, which can be for patients as well as for health care professionals. Excluding these apps, the rest have different purposes depending on the disease. Hence, developers have tried to take advantage of the characteristics of each illness. They centered on prevention in diseases with no cure or with a cure based on medications where no mobile phone aid is needed such as Alzheimer and dementias, with games useful for their prevention; and malaria, with mosquito-repellant apps. There are also several apps for HIV/AIDS focused on the prevention with educational apps for the public (and others for patients). Health care professionals are the objective of the apps designed for LRI, stroke, and lung cancer, which offer aid in the diagnosis or the assessment of the stage/status of the diseases. Finally, in the cases of diarrhea and COPD the majority of apps (excluding the informative ones) are designed for treatment: personal remedies for diarrhea (mild diarrheas), and COPD trackers as well as educational apps for the use of inhalers.

Finally, the enormous difference in the number of applications found in the distinct stores is surprising. Google play and iTunes are clearly the ones with more apps followed by far by Windows Phone Apps+Games and BlackBerry World, as previously observed [71]. The case of BlackBerry is unusual: in November 2012, it had a market share of 7.3% [72], but it barely has applications for the diseases searched. On the other hand, Microsoft Phone had less market share (3% in November 2012 [72]) but more apps, yet much less than the number of apps of Google play and iTunes. As a result, a great number of users of BlackBerry and Windows Phone would abandon their phones, changing them for others that have the maximum number of apps available. Indeed, this is already happening [72] and can be a reason for the lack of apps for Windows Phone and BlackBerry, because developers are discouraged from creating apps for these stores.

For future work, several things can be done. It is possible to develop a mobile app for LRI because of the absence of apps related to it and its importance in mortality (third cause of death worldwide). It can include the most common infections such as bronchitis or pneumonia. Another possibility is to fill the empty space in mobile apps related to the trachea, bronchus, and lung cancers, for example developing an assistive and informative application for aiding patients who are being treated for these cancers.

## Abbreviations

COPD: chronic obstructive pulmonary disease
CVD: cardiovascular disease
DALY: disability-adjusted life year
HIV/AIDS: human immunodeficiency virus /acquired immunodeficiency syndrome
IHD: ischemic heart disease
LRI: lower respiratory infections
NIH: National Institutes of Health
PDA: personal digital assistant
RSV: respiratory syncytial virus
WHO: World Health Organization
